# Regulators of proteostasis are translationally repressed in fibroblasts from patients with sporadic and LRRK2-G2019S Parkinson’s disease

**DOI:** 10.1038/s41531-023-00460-w

**Published:** 2023-02-06

**Authors:** Dani Flinkman, Ye Hong, Jelena Gnjatovic, Prasannakumar Deshpande, Zsuzsanna Ortutay, Sirkku Peltonen, Valtteri Kaasinen, Peter James, Eleanor Coffey

**Affiliations:** 1 Turku Bioscience Centre, University of Turku and Åbo Akademi University, Turku, Finland; 2 Lund University, Department of Immunotechnology, Lund, Sweden; 3 Department of Dermatology, University of Turku and Turku University Turku University Hospital, Turku, Finland; 4 Department of Dermatology and Venereology, University of Gothenburg, and Sahlgrenska University Hospital, Gothenburg, Sweden; 5 Clinical Neurosciences, Faculty of Medicine, University of Turku, Turku, Finland; 6 Neurocenter, Turku University Hospital, Turku, Finland

**Keywords:** Parkinson's disease, Proteins, Sequencing

## Abstract

Deficits in protein synthesis are associated with Parkinson’s disease (PD). However, it is not known which proteins are affected or if there are synthesis differences between patients with sporadic and Leucine-Rich Repeat Kinase 2 (LRRK2) G2019S PD, the most common monogenic form. Here we used bio-orthogonal non-canonical amino acid tagging for global analysis of newly translated proteins in fibroblasts from sporadic and LRKK2-G2019S patients. Quantitative proteomic analysis revealed that several nascent proteins were reduced in PD samples compared to healthy without any significant change in mRNA levels. Using targeted proteomics, we validated which of these proteins remained dysregulated at the static proteome level and found that regulators of endo-lysosomal sorting, mRNA processing and components of the translation machinery remained low. These proteins included autophagy-related protein 9A (ATG9A) and translational stability regulator YTH N6-ethyladenosine RNA binding protein 3 (YTHDF3). Notably, 77% of the affected proteins in sporadic patients were also repressed in LRRK2-G2019S patients (False discovery rate (FDR) < 0.05) in both sporadic and LRRK2-G2019S samples. This analysis of nascent proteomes from PD patient skin cells reveals that regulators of proteostasis are repressed in both sporadic and LRRK2-G2019S PD.

## Introduction

Parkinson’s disease (PD) is the fastest-growing neurodegenerative disease, with an estimated 6.1 million patients worldwide in 2016^1^. The cardinal motor symptoms include bradykinesia, muscular rigidity, resting tremor, and postural instability, but the disease is often complicated by non-motor symptoms, such as hyposomia, constipation, and REM sleep behavior disorder, which can precede motor disease onset by years^2^. However, the disease is heterogenous, and phenotypes of motor and non-motor symptoms together with general disease trajectories differ broadly among patients^3^. Moreover, the diagnosis itself is error prone as the core motor symptoms can be difficult to distinguish from those of other movement disorders^4^. There is a growing interest in exploiting ‘omic’ technologies to understand the molecular changes that contribute to PD pathology and its heterogeneity, and to identify markers that could potentially assist in the diagnosis and stratification of patients, ideally at an early stage^5,6^.

Emerging evidence from cellular and rodent models as well as patient-induced pluripotent cells suggests that protein synthesis is deregulated in PD^7–11^. We have found that de novo protein synthesis is reduced in fibroblasts from PD patients with a receiver operating characteristic area under the curve of 0.925, suggesting good predictive power of this function as a biomarker readout^12^. Moreover, fibroblasts derived from patient skin biopsies have been shown to harbor disease-associated features of PD. For instance, alpha-synuclein seeding activity measured from PD cadaver skin was shown to successfully distinguish patients with synucleinopathies^13–15^, and sebum from PD patients was shown to contain disease-defining lipid biomarkers^16^. Interestingly also, in a recent study of organoids derived from PD patient fibroblasts, “cytoplasmic ribosomal proteins” and “translation factors” were the most enriched transcriptomic features^17^. This points to the suitability of patient fibroblasts as a useful source of biopsy material for molecular understanding of PD as well as biomarker identification.

Here we set out to identify which proteins were deregulated at the level of mRNA translation in PD patient cells using bio-orthogonal non-canonical amino acid tagging (BONCAT)^18^. This method uses proteomics to identify nascent proteins that have been purified on the basis of a non-canonical amino acid tag that is introduced to the cells during a pulse labeling. We examined nascent proteins from fibroblasts isolated from the sporadic PD cases and healthy volunteers in Southwest Finland, and from cells of patients carrying a gain of function LRRK2-G2019S mutation, obtained from the National Institute of Neurological Disorders (NINDS) and Telethon Network of Genetic Biobanks (TNGB) repositories. From the resulting newly synthesized proteome, we found that certain polypeptides were less abundant in PD groups compared to healthy. Among these, 15 proteins remained significantly changed (FDR < 0.05) at the level of the whole cell proteome without any regulation of the corresponding mRNA transcripts. These proteins are known regulators of endo-lysosomal sorting, mRNA processing and of protein synthesis itself.

## Results

### Fluorescent non-canonical amino acid tagging (FUNCAT) reveals that nascent protein synthesis is reduced in fibroblasts from sporadic and LRRK2-G2019S Parkinson’s patients

**Fig. 1:**
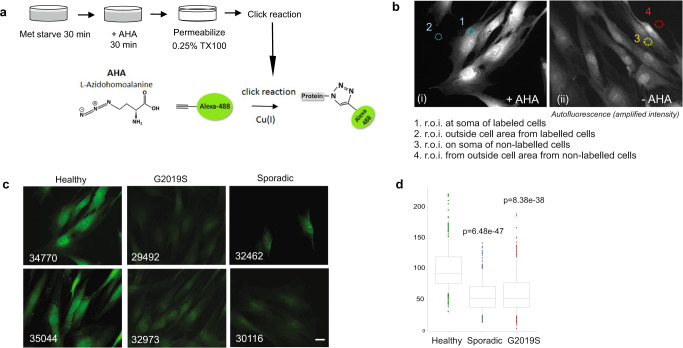
Bulk protein synthesis is reduced in Parkinson’s disease patient fibroblasts. **a** Schematic of metabolic labeling. **b** Scheme illustrating how regions of interest (r.o.i.) were positioned for measuring soma and background fluorescent intensities. Representative images of cells with (+AHA) and without AHA (-AHA) label are shown (i, ii). The brightness of image i is enhanced in order to see the autofluorescence which would otherwise not be visible by eye. Image acquisition settings were constant throughout. **c** Representative images of AHA-labeled fibroblast cells from G2019S or sporadic PD patients or healthy controls and their NINDS repository ID are shown. Scale indicates 20 µm. **d** Mean fluorescence intensities after background and autofluorescence intensity subtraction are shown from healthy, G2019S and sporadic cases. Adjusted *p*-values were calculated using Student’s *t*-test and corrected with Benjamini and Hochberg method. Box plots depict 50% of middle values from bottom 25% percentile to top 75% percentile, with a line showing the median values. Whiskers show values at 1.5 times the interquartile range.

In order to validate our earlier finding that translation is decreased in PD patient cells, we grew non-transformed fibroblasts from patients carrying a LRRK2-G2019S mutation or those diagnosed with sporadic PD, and healthy donors. Patient details (age, gender etc.) are described in Table 1. To label nascent proteins, cells were metabolically labeled according to the FUNCAT method as previously described^12,19^. In this procedure, endogenous methionine is replaced with the L-Azidohomoalanine (AHA) analog of methionine. AHA-labeled proteins were then tagged with Alexa-488-alkyne by a cycloaddition *click* reaction (Fig. 1a). This allowed quantitative fluorescence measurement of *de novo* synthesized proteins according to the scheme (Fig. 1b). As previously, we found that Alexa-488 fluorescence was significantly reduced in PD samples compared to control cells indicating that bulk *de novo* synthesis was reduced (Fig. 1c, d). Raw data can be found in Supplementary Table 1. Nascent protein synthesis was reduced in both G2019S carriers and sporadic patients alike.

**Table 1:** Demographic and clinical characteristics of patients and healthy controls.

Cell I.D.	Description	Source	Gender	Age at sampling	Estimated age of onset	Motor symptom duration (years)	Hoehn & Yahr stage	Motor MDS-UPDRS (Part III)^a^	DAT binding defect with[123I]FP-CIT	Measured in experiment
PD1	Sporadic	T.U.H.	M	60	56	4	2	50	Not performed	A,P,W,Q
PD2	Sporadic	T.U.H.	M	69	60	9	2	42	Not performed	A,P,W,Q
PD3	Sporadic	T.U.H.	M	59	49	10	3	57	yes	A,P,W,Q
PD4	Sporadic	T.U.H.	M	52	50	1.5	1	25	Not performed	A,P,W,Q
PD5	Sporadic	T.U.H.	M	64	59	5	2	30	Not performed	A,P,W,Q
PD6	Sporadic	T.U.H.	M	65	61	4	2	37	yes	A†,P,W,Q
PD7	Sporadic	T.U.H.	M	69	58	11	2	25	Not performed	A,P,W,Q
PD8	Sporadic	T.U.H.	M	68	65	3	2	32	yes	P,Q
PD9	Sporadic	T.U.H.	M	66	64	2	2	32	yes	A,P,Q
PD10	Sporadic	T.U.H.	M	49	46	3	1	26	yes	A,P,W,Q
PD11	Sporadic	T.U.H.	M	60	50	10	3	47	yes	P,Q
PD12	Sporadic	T.U.H.	F	66	56	10	2	15	yes	A,P,Q
PD13	Sporadic	T.U.H.	M	63	48	15	3	20	Not performed	A,P,Q
29492	LRRK2 G2019S	NINDS	M	72	58	14	2	–	–	A,P
32949	LRRK2 G2019S	NINDS	M	81	75	6	1,5	14	–	A,P
FF0212011	LRRK2 G2019S	EMB	M	47	38	9	–	–	–	A,P
FF0432012	LRRK2 G2019S	EMB	M	57	54	3	–	–	–	A,P^b^
FF0642009	LRRK2 G2019S	EMB	F	58	41	17	–	–	–	A,P
C1	Healthy subject	T.U.H.	F	57	–	–	–	–	–	A,P,W,Q
C2	Healthy subject	T.U.H.	F	54	–	–	–	–	–	A,P,W,Q
C3	Healthy subject	T.U.H.	F	66	–	–	–	–	–	A,P,Q
C4	Healthy subject	T.U.H.	M	58	–	–	–	–	–	A,P,W^c^,Q
C5	Healthy subject	T.U.H.	F	49	–	–	–	–	–	P,W,Q
C6	Healthy subject	T.U.H.	M	73	–	–	–	–	–	A,P,W,Q
C7	Healthy subject	T.U.H.	F	65	–	–	–	–	–	A,P,W,Q
34770	Healthy subject	NINDS	M	72	–	–	–	–	–	A,P
36320	Healthy subject	NINDS	F	71	–	–	–	–	–	A,P
35044	Healthy subject	NINDS	M	77	–	–	–	–	–	A,P
F0172014	Healthy subject	EMB	F	68	–	–	–	–	–	P
F0492012	Healthy subject	EMB	M	41	–	–	–	–	–	A,P
F0502011	Healthy subject	EMB	F	63	–	–	–	–	–	A,P
F0682013	Healthy subject	EMB	F	58	–	–	–	–	–	A,P
Average Sporadic	–	–	–	62.3	55.5	6.7	2.1	33.7	–	–
Average G2019S	–	–	–	64.5	53.2	9.8	2.0	14.0	–	–
Average Healthy	–	–	–	62.3	–	–	–	–	–	–

Fibroblasts from PD (PD1–PD13) patients with sporadic disease, or healthy donors (C1–C7) from Turku University Hospital (TUH) are shown alongside LRRK2-G2019S PD patient and healthy subject fibroblasts from the National Institute of Neurological Disorders (NINDS) and the TNGB. Gender, male (m) or female (f) is indicated and “age” at the time of biopsy is shown.

^a^Unified Parkinson’s Disease rating scale (UPDRS). All 13 patients from TUH had received antiparkinsonian pharmacotherapy by the time of biopsy. Five patients were receiving levodopa (PD2, PD3, PD7, PD11, and PD13). Some of these were also receiving MAO-B inhibitors and/or dopamine agonists. The rest were receiving MAO-B inhibitors and/or dopamine agonists without levodopa. Measured in experiment: A = AHA experiment, P = PRM, Q = qPCR, W = WB.

^b^C5 and PD 6 in AHA and 432012 in PRM were measured, but were outliers based on lower intensity.

^c^C4 served as a control sample for western blotting.

### Isolation and LC-MS/MS identification of newly synthesized proteins from LRRK2-G2019S carriers and sporadic patients

**Fig. 2:**
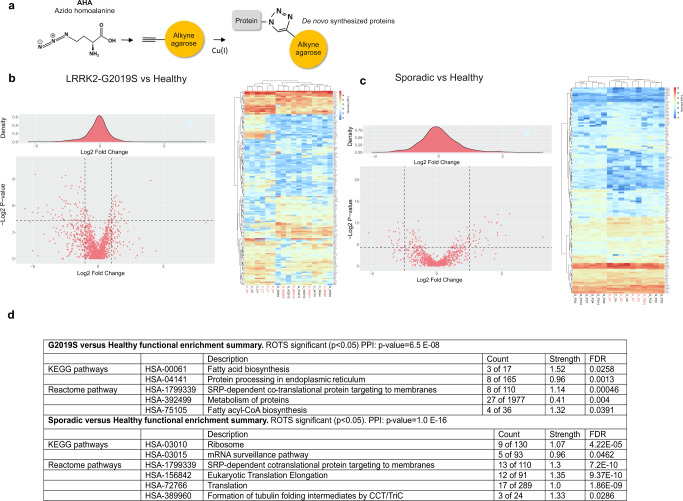
De novo protein synthesis is altered in LRRK2-G2019S and sporadic PD patient fibroblasts versus healthy controls. **a** Schematic overview of click reaction used to isolate newly synthesized proteins. **b** Volcano plot of all AHA-labeled protein intensities for LRKK2-G2019S versus healthy. ROTS *p*-values are depicted. Heat-map with K-means hierarchical clustering depicts the significantly regulated nascent protein intensities in fibroblasts from LRRK2-G2019S patients versus healthy individuals. The union of ROTS and *t*-test with *p*-value < 0.05 are shown. Red sample label indicates female gender. Proteins are labeled with their corresponding gene names as they are more concise. **c** Volcano plot of all AHA-labeled protein intensities for sporadic versus healthy using ROTS statistical test, and heatmap with hierarchical clustering depicts the nascent proteins as in **b** above. **d** Proteins with intensity fold change > 1.5 and ROTS *p*-value < 0.05 were analyzed in STRING. Top ranking (by FDR or count in network) functional enrichments for KEGG pathways and Reactome pathways are shown. Strength is calculated from log_10_(observed/expected) network proteins. FDR shows *p*-values corrected for multiple testing using the Benjamini-Hochberg procedure.

We set out to identify which mRNA transcripts were differentially regulated at the level of translation in PD patient cells, investigating both sporadic and LRRK2-G2019S cases. Skin punches were isolated from 10 sporadic PD cases and 6 healthy donors from patients attending TUH and from an accompanying family member (usually a spouse or sibling), as previously described^12^. In addition, we analyzed five fibroblast samples from LRRK-G2019S patients and six from healthy controls using material from the NINDS and TNGB repositories. Details of subjects including age and sex are described in Table 1. We used the BONCAT method^18^ (Fig. 2a), to isolate newly translated proteins from patient and healthy donor fibroblasts followed by mass spectrometry analysis. Data was analysed and preprocessed using MaxQuant and Perseus analysis software and multiple statistical tests were done using the PhosPiR proteomic and phosphoproteomic data analysis pipeline^20^. The sum of peptide intensities for newly synthesized proteins from LRRK2-G2019S and sporadic patients versus healthy are shown in Fig. 2b and c respectively. Raw and statistically analyzed data can be found in Supplementary Table 2. Protein intensities were overall lower in cells from PD individuals compared to healthy, regardless of whether they were from LRRK2-G2019S or sporadic patients. Synthesis of 30 nascent proteins was reduced in LRRK2-G2019S cells compared to healthy controls and 33 were significantly decreased in sporadic versus healthy (Fig. 2b, c, supplementary Table 2). There was 65% overlap in the identity of nascent proteins that changed significantly compared to controls in both LRRK2-G2019S and sporadic groups (*p* < 0.05 in ROTS), suggesting a degree of mechanistic overlap.

We next carried out an enrichment analysis of nascent proteins that were significantly (ROTS *p*-value < 0.05) altered in LRRK2-G2019S or sporadic patient cells using the STRING database^21^. This identified that the KEGG pathways linked to *fatty acid biosynthesis* and *Signal Recognition Particle-dependent co-translational protein targeting to membrane* were functionally enriched in the nascent proteome from LRRK2-G2019S and sporadic patient fibroblasts versus healthy with FDRs of 0.0258 and 0.0013 respectively (Fig. 2d). This STRING process incorporates components that control the translation of proteins destined for the secretory pathway and their translocation to the endoplasmic reticulum. In contrast, in sporadic PD, KEGG pathway “*Ribosom*e” (FDR = 4.22E−05) and Reactome pathway by network count“*Formation of tubulin folding intermediates by CCT/Tric*” (Count = 3/24), were most significantly enriched (Fig. 2d). Mutant^22,23^ and active LRRK2 is known to associate with microtubules and regulate trafficking^24,25^, and this disturbance in the homeostasis of this network may be LRRK2 dependent.

### Several nascent proteome changes in LRRK2-G2019S and sporadic PD fibroblasts remain altered at the total proteome level

**Fig. 3:**
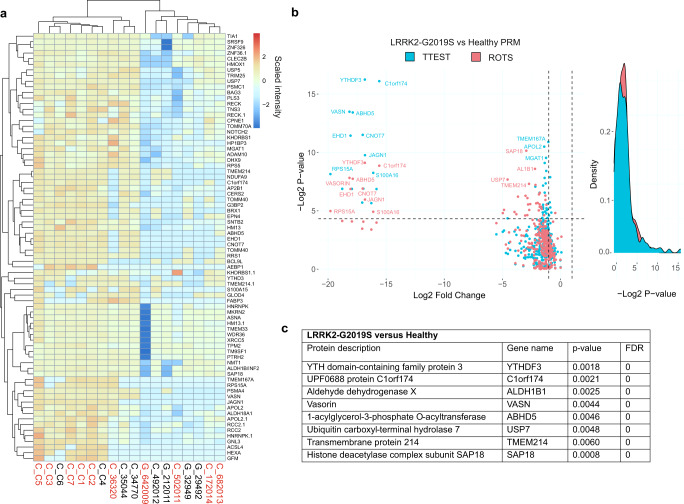
PRM validation of protein homeostasis changes in LRRK2-G2019S patient cells. Targeted proteomics (PRM quantification of peptides from Fig. 2b) was carried out to validate protein intensity differences observed from AHA-labeled, de novo synthesized proteins from total lysates of patient cells. **a** Hierarchical clustering of PRM results shows overall protein intensities in fibroblasts from LRRK2-G2019S and healthy controls. Proteins with *p*-value < 0.05 from either ROTS and/or t-test are shown. **b** Volcano plot of PRM results from LRRK2-G2019S vs healthy. *P*-values from ROTS and *t*-test statistical tests are shown. Red sample label indicates female gender. **c** The top changing proteins with FDR = 0 from ROTS test are described.

**Fig. 4:**
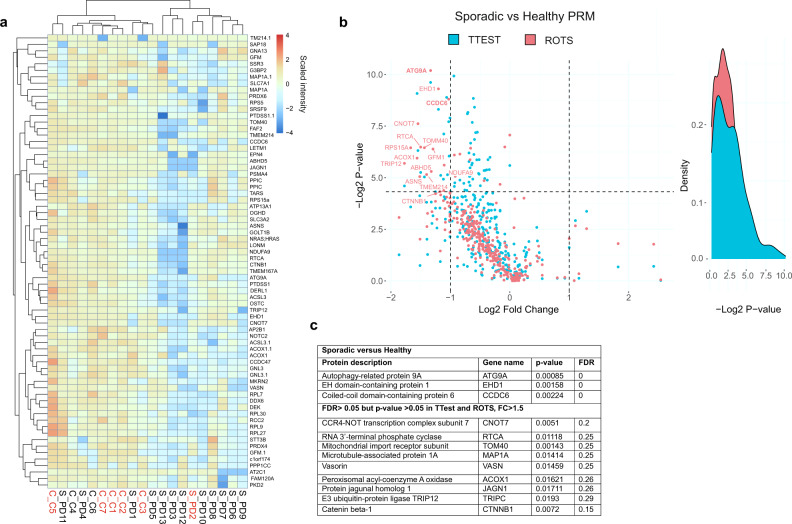
PRM validation of protein homeostasis changes in sporadic Parkinson’s patient cells. Targeted proteomics (PRM quantification of peptides from Fig. 2c), was carried out to validate protein intensity differences from total lysates of patient cells. **a** Hierarchical clustering of PRM results shows overall protein intensities in fibroblasts from sporadic and healthy controls with a *p*-value < 0.05 in either ROTS and/or *t*-test. **b** Volcano plot of PRM results from sporadic vs healthy. *P*-values from ROTS and *t*-test statistical tests are shown. Red sample label indicates female gender. **c** The top changing proteins are described.

We next set out to identify which among the deregulated nascent proteins were disturbed at the level of the total static proteome. For this, we used targeted proteomics and screened for a list of peptides (Supplementary Table 2) that included unique identifier peptides for each proteins that changed significantly in the discovery phase analysis (Fig. 2). Significant changes were defined from group comparisons (healthy versus PD and healthy versus G2019S) according to four distinct MS/MS data preprocessing steps (MaxQuant raw intensity values, LFQ normalized data with missing value either imputed using Perseus software or replaced by one). This inclusive approach was taken to allow for relative strengths of different pre-processing methods, and to enable us to evaluate the prediction accuracy of the preprocessing methods themselves. The union list of all significantly changing proteins for G2019S versus healthy and sporadic versus healthy, and all PD versus healthy incorporated 251 proteins in all (Supplementary Table 2). The results of the PRM-analysis for healthy versus LRRK2-G2019S and sporadic groups are shown as volcano plots and heatmaps in Figs. 3 and 4 respectively. The raw and statistical data can be found in Supplementary Table 2).

Interestingly, all nascent polypeptides that were significantly altered in the LRRK2-G2019S patient samples versus healthy, were significantly reduced in total cell lysates, using PRM analysis. The most robustly decreased were YTHDF3, Chromosome 1 open reading frame 174 (C1orf174), Vasorin (VASN), Abhydrolase domain containing 5, lysophosphatidic Acid Acyltransferase (ABHD5), EH domain-containing protein 1 (EHD1), CCR-NOT transcription complex subunit 7 (CNOT7), Jagunal homolog 1 (JAGN1), ribosomal protein 15A (RPS15A) and S100 calcium binding protein A16 (S100A16) (Fig. 3a–c). In sporadic patient samples, the PRM analysis also identified solely decreased protein intensities relative to healthy donors (Fig. 4a–c, supplementary Table 2), although the extent of decrease was reduced compared to the LRRK2-G2019S patient group.

### There is a substantial overlap in the deregulated nascent proteome from sporadic and G2019S Parkinson’s patient fibroblasts

**Fig. 5:**
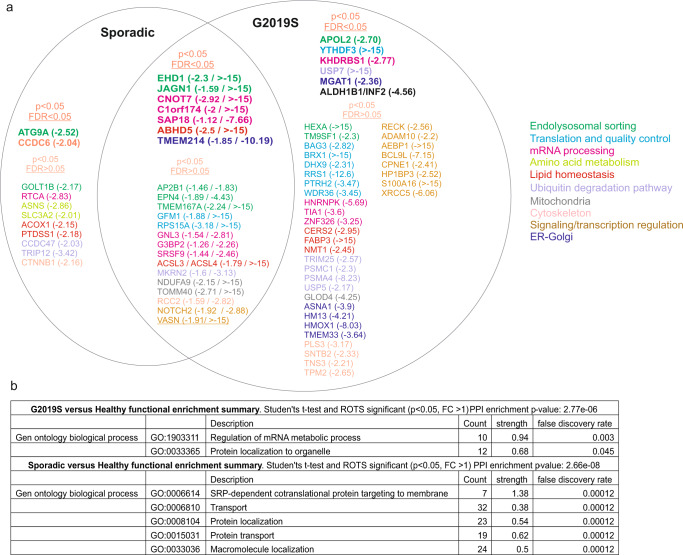
A. Venn diagram summary of proteins that show reduced homeostasis in LRRK2-G2019S and sporadic PD patients. **a** Proteins (indicated by gene names) are grouped according to significance. For each group comparison, proteins that have a fold change >1.5 and a *P* < 0.05 and FDR < 0.05 in either ROTS or *t*-test are shown in a separate category to proteins with *p* < 0.05 but FDR > 0.05 in either test. Proteins plotted in the overlap region are significantly changing in both sporadic and LRRK2-G2019S groups, where FDR < 0.05 in either ROTS or t-test in either group and *p*-value < 0.05 in the other group. **b** STRING enrichment analysis of the proteins shown in **a**. according to LRRK2-G2019S and sporadic patient groups. Functional enrichments for biological process, local network clusters and KEGG pathways are shown. Input data is from the union of ROTS and *t*-test PRM analysis changes (*p* < 0.05 and FC > 1). Strength is calculated from log10(observed/expected) network proteins. FDR values show *p*-values corrected for multiple testing using the Benjamini-Hochberg procedure.

Whether LRRK2-G2019S PD and sporadic PD represent molecularly distinct or overlapping forms of the disease is of interest for treatment development^12,26,27^. We therefore examined the overlap in proteins between LRRK2-G2019S PD and sporadic PD (Fig. 5a). In the sporadic group, 77% of deregulated proteins (FDR < 0.05) overlapped with the LRRK2-G2019S group with the exception of the autophagy-related protein 9A (ATG9A) and coiled-coil domain-containing protein 6 (CCDC6) which were only found to be significantly altered in sporadic patients (Fig. 5a). ATG9A plays an essential role in autophagy (where it forms the pre-autophagosomal structure assembly site), and in vacuole transport vesicle formation^28^. In LRRK2-G2019S patients, 53% of downregulated proteins (FDR < 0.05) overlapped with the sporadic group. The protein that was most highly downregulated was YTHDF3, which was unique to this patient group. This protein belongs to a family of N6-methyladenosine RNA modification readers that can recruit molecular complexes to M^6^A sites, to impact RNA splicing, export, stability, trafficking, and translation efficiency^29^. Consistent with this, functional enrichment analysis of the LRRK2-G2019S patient data using all proteins with *p* < 0.05 identified the *Regulation of mRNA metabolic process and protein localization to organelle* (GO:1903311 and GO:0033365) as the most significantly enriched gene ontology. In contrast, in the sporadic group, *SRP-dependent cotranslational protein targeting to membrane was the most significant term followed by Transport, and protein localization* (GO:0006614 and G:006810) (Fig. 5b).

### mRNA levels of altered nascent proteins do not significantly change in patient samples

**Fig. 6:**
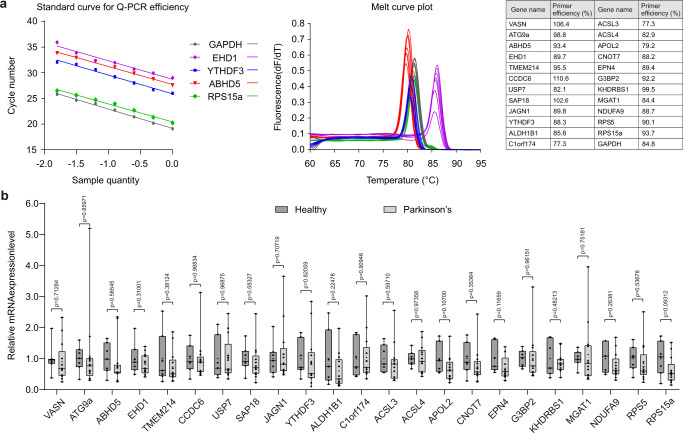
mRNA levels of translationally repressed proteins do not change in sporadic patient fibroblasts. **a** Sample standard curves for primer efficiencies are shown including a table of all primer efficiencies. All primer pairs amplified a product with a single melting temperature peak, confirming the amplification of a single specific product (representative data shown). Resulting primer efficiencies were all in the range of 75–110% qPCR reaction efficiency. **b** The relative expression of mRNA was measured for the 23 genes, the protein counterparts of which significantly changed in PD patients with an FDR < 0.05 in either ROTS or *t*-test. GAPDH was included as a housekeeping gene. Whiskers extend from the smallest to the largest values, with a box depicting 50% of middle values from bottom 25% to top 75% and line shows the median. Two-tailed Student’s *t*-test did not reveal significant changes in the gene expression among patient versus healthy in any of the samples.

To examine whether the observed changes in proteins were due to altered mRNA levels, we carried out quantitative PCR (qPCR) on the transcripts of the protein-coding genes that changed (FDR < 0.05) using either the ROTS or *t*-test in Fig. 5. Primer efficiencies and melt curves are shown (Fig. 6a and in Supplementary Table 3 and Supplementary Fig. 1). Relative mRNA levels were quantified using the Pfaffl method which accounts for differences in primer efficiencies (Fig. 6b and Supplementary Table 3)^30^. Although there were minor trends towards decreased levels in patients versus healthy, there were no significant differences for any of the 23 mRNAs measured. This result is supported by a previous meta-analysis study of RNAseq data from PD patient fibroblasts where the same genes were also not significantly altered^31^.

### Translationally repressed proteins are rescued upon treatment with LRRK2 inhibitor

**Fig. 7:**
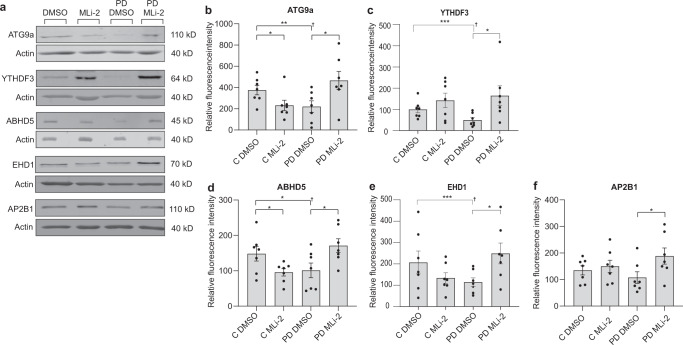
Translationally repressed protein levels are recovered upon treatment with LRRK2 inhibitor. **a** Representative western blots of TUH healthy and sporadic PD fibroblasts treated with MLi-2 (100 nM) or DMSO for 16 h as indicated. **b**–**f** Quantification results from fluorescence detection of proteins by immunoblotting is shown for ATG9A, YTHDF3, ABHD5, EHD1, and AP2B1 from healthy and sporadic PD patient fibroblasts. Normalized data and SEM are shown. To determine if these proteins decreased compared to baseline (calculated from the average of all healthy samples *p*-value †), a one-tailed *t*-test was used.

While PRM is considered to be a reproducible, specific, and quantitatively accurate method^32^, mass spectrometry systems are not readily available to every lab or clinic. Therefore, we tested to what extent these changes could be observed using an antibody-based method. We immunoblotted fibroblast lysates from healthy subjects and sporadic PD patients for five of the significantly altered proteins from the PRM analysis (FDR < 0.05); ATG9A, YTHDF3, ABHD5, EHD1, and AP2B1 (Fig. 7, Supplementary Table 4). This included cells from five healthy individuals and seven sporadic PD cases from TUH cohort (Table 1), treated in vitro with or without 100 nM of LRRK2 inhibitor MLi-2 to determine if the changes observed were LRRK2 dependent. We found that ATG9A, YTHDF3, EHD1, and ABHD5 were significantly downregulated in PD samples when compared to healthy, while AP2B1 showed a trend towards decreased expression (Fig. 7a–f). Interestingly, although significant, the magnitude of responses were somewhat lower using western blotting compared to mass spectrometry. This could be due to several reasons. Firstly, targeted mass spectrometry is more quantitative than western blotting^33^ and because the data is matched to a reference peptide, there is no ambiguity regarding the actual protein identified, which is not the case for western blotting. Moreover, mass spectrometry distinguishes proteoforms and homologs, whereas in western blotting there is not a similar level of certainty even with monoclonal antibodies.

To test whether the deregulation of these proteins was LRRK2 dependent, we treated cells with or without MLi-2. In every case, LRRK2 inhibitor rescued the downregulation of ATG9A, YTHDF3, EHD1, ABHD5, and AP2B1 in PD samples (Fig. 7a–f). Interestingly, treatment of healthy cells with MLi-2 alone significantly decreased expression of ATG9A and ABHD5. This may represent a proteostatic mechanism whereby the cell decreases the levels of these proteins to compensate for the increased translation upon LRRK2 inhibition. It is in that case unclear why only these two proteins would be affected. While this may not be a long-lasting effect, it is worth noting as it may not be a desirable in a clinical setting. In conclusion, this analysis identified proteins that are repressed at the level of translation in PD patient fibroblasts. These could potentially provide useful information on disease mechanism and/or serve as markers for treatments based on inhibition of LRRK2.

## Discussion

A growing number of studies in patient-derived cells and microphysiological systems have linked the cellular process of mRNA translation with PD^10,12,17^. In this study, we use PD patient fibroblasts to mine the Parkinson’s translatome. This identifies that protein regulators of proteostasis are translationally repressed. Proteostasis encompasses several cellular processes including biogenesis, folding, trafficking, and degradation of proteins, all contributing to the overall maintenance of the proteome^7^. Notably, 77% of the affected polypeptides (FDR < 0.05) in sporadic patients are also downregulated in LRRK2-G2019S PD, indicating that the underlying mechanism is at least partly shared in both types of PD (Fig. 5a). These translationally repressed proteins are involved in endolysosomal sorting (ATG9A, EHD1, JAGN1 and APOL2); mRNA processing (CNOT7, C1orf174, SAP18, and KHDRBS1); and in the translation mechanism itself (YTHDF3, RP15A, RPS5, GFM1, RRS1, and BRX1). Accordingly, these same functions are enriched in the STRING gene ontology analysis, where a higher than expected level of protein:protein interactions are identified among the translationally repressed proteins (Fig. 5). It is already known from cell and animal models of PD that LRRK2 regulates endolysosomal function, translation and autophagy^34,35^; here we identify the dysregulation of these functions in clinical samples. Specifically, we identify which nascent proteins are affected and which are not recovered by homeostatic mechanisms but remain low at the total proteome level, and are therefore most likely to contribute to the underlying pathology. Accordingly, several of the affected polypeptides identified are already implicated in PD. These are discussed in detail below.

The top downregulated proteins in sporadic patient fibroblasts are ATG9A and EHD1, which regulate endosomal sorting at the level of retromer formation^36^, as well as CCDC6, which was recently associated with Alzheimer’s disease^37^. Autophagy is the process whereby cells dispose of aggregated or misfolded proteins via lysosomal degradation, a deficiency in which contributes to the build-up of alpha-synuclein aggregates^38^. ATG9A is needed to supply new membrane during autophagosome formation and maturation^39–41^, while it is also implicated in the innate immune response^42^. ATG9A has previously emerged in the context of PD where in patients carrying the D620- > N mutation in the vacuolar sorting-associated protein 35 (VSP35), ATG9A trafficking was shown to be impaired^43^. Here we find that ATG9A levels are significantly lower in sporadic patient fibroblasts and although they are up to 11-fold lower in G2019S patients, there is no significant change overall in this group, possibly due to the smaller number of patient samples available carrying this mutation. Given that ATG9A is required for autophagosome formation, its translational repression in PD is very interesting and could potentially explain how LRRK2 suppresses clearance of alpha-synuclein leading to its aggregation in PD^39,44^. Also, even though these results are from skin cells, fibroblasts originate from the same embryonic stem cell layer as neural tissue, and show some overlapping gene expression with brain, thus fibroblasts are arguably relevant for the study of PD^45^. Moreover, autophagic flux is also decreased in fibroblasts from LRRK2-G2019S patients^46,47^, and we show that translational repression of ATG9A is LRRK2-dependent (Fig. 7). In brain, loss of ATG9A is known to induce lesions specifically in axons^48^, thus repression of ATG9A levels in PD could contribute to dopaminergic neuron atrophy^49,50^. Further linking to axonal degeneration, we find that proteins involved in synaptic endocytosis, AP2B1, EPN4, and EHD1 are significantly downregulated in sporadic and G2019S patient cells. This may also be relevant for axonal degeneration in PD^51^. Among these, it is already known that LRRK2 interacts AP2B1^52^, a critical component of clathrin-mediated endocytosis.

In the LRRK2-G2019S cohort, YTHDF3 is the most strongly downregulated protein using mass spectrometry detection (Fig. 5), whereas YTHDF3 also decreases in sporadic cases by immunoblotting (Fig. 7). YTHDF3 recognizes the common M6-Methyladenosine modification of mRNA which acts to promote mRNA splicing and stabilization of translation^53^. Downregulation of YTHDF3 is therefore expected to destabilize mRNAs and to directly reduce translation. YTHDF3 repression could in principle explain several of the disturbed cellular mechanisms associated with PD. Interestingly therefore, components of the translation machinery were also reduced (Fig. 5).

Biomarker monitoring of PD and of LRRK2 activity in PD is important for clinical advancement in this area. One of the potential biomarkers currently studied is phospho-Ras-related protein Rab-10 (Rab10). However, although phospho-Rab10 is readily dephosphorylated in patient cells upon LRRK2 inhibition^54,55^, there are conflicting results regarding its ability to differentiate between PD and healthy individuals for example^55–58^. Moreover, biomarkers are still lacking that would discriminate between PD and atypical parkinsonian disorders. Some of the proteins that we identify in this study have been previously associated with PD. For example, we show that long-chain-fatty-acid-CoA ligase 4 (ACSL4) is downregulated at the translatome and proteome level. ACSL4 regulates lipid composition and ferroptosis which results from accumulation of lipid peroxides, and is one of the pathogenic mechanisms in PD^59^. Upregulation of ACSL4 has been shown to rescue MPTP-induced motor deficits and dopaminergic neuron loss in the substantia nigra, by reducing iron aggregation^60^. The ribosomal protein RPS15A is also downregulated at the level of translatome in our analysis. This protein has been shown to be downregulated in the substantia nigra of PD patients^61^. Moreover, TOMM40 which we find is downregulated in PD, is also decreased in PD patient brain and contributes to mitochondrial dysfunction induced by alpha-synuclein accumulation^62^. TRIP12, which is also downregulated in our patient samples, interacts with and ubiquitinates the PD-associated glucocerebrosidase^63^. The overall effect of downregulation of TRIP12 might lead to dysregulation of ubiquitination, thought to be protective in PD, and indeed we see other components of this system MKRN2, PSMA4, PSMC1, TRIM25, USP5, and 7 downregulated in our samples. Finally, NOTCH2 which we identify here as downregulated at the level of translation, is a proposed biomarker for depression associated with PD^64^. While all deregulated proteins identified in this study represent potential biomarkers, independent validation and further testing would be necessary.

We find that a number of the reduced polypeptides in patient cells respond to LRRK2 kinase inhibition. Indeed, for every target tested (ATG9A, YTHDF3, EHD1, ABHD5, and AP2B1) there is a PD-specific rescue of homeostasis following treatment with LRRK2 inhibitor (Fig. 7). As discussed above it is notable that several of the downregulated proteins are known to be altered in PD brain, suggesting that they may be involved in the pathological mechanism. This makes them potentially interesting clinical targets and biomarkers for PD drug trials and treatment monitoring. Nonetheless, further studies would be needed to clarify whether these changes are specific for PD, or whether they are also relevant to other neurodegenerative diseases such as Alzheimer’s disease for example.

In summary, by investigating the nascent proteome from PD patient fibroblasts, we identified that key regulators of autophagy, translation and endosomal sorting are reduced in sporadic and LRRK2-G2019S patients alike. These findings support the growing evidence for deregulated proteostasis in PD and highlight potential new therapeutic targets and biomarkers.

## Methods

### Cultivation of fibroblasts from patient skin biopsies

Skin punches were taken from the upper arm of donors and PD patients by a licensed dermatologist at Turku University Hospital (TUH). Punches were placed immediately in Minimal essential medium (M.E.M., Sigma Aldrich). Tissue was chopped into several small pieces using a sterile blade and incubated in M.E.M. supplemented with Gln (2 mM), penicillin (50 U/ml) and streptomycin (50 μg/ml) at 37 °C, 5% CO_2_. Fibroblasts were passaged when 95% confluent. Experiments were done at passage 7–14. Patient and control skin fibroblasts from the NINDS cohort were from the NINDS Cell Line Repository (http://ccr.coriell.org/ninds). Patient and control skin fibro-blasts from the TNGB cohort were from the “Cell Line and DNA Biobank from Patients Affected by Genetic Diseases,” member of the Telethon Network of Genetic Biobanks (project no. GTB12001), funded by Telethon Italy.

### Description of clinical samples

The fibroblasts used in this study came from three sources. All sporadic cases were from TUH. These included 13 sporadic PD patients and seven age-matched healthy cases. These patients were diagnosed as having PD by neurological testing by a movement disorder specialist using Movement Disorder Society (MDS) clinical diagnostic criteria for PD combined with dopamine transporter single-photon emission computed tomography (SPECT) imaging with [^123^I]FP-CIT as tracer for seven patients. For LRRK2-G2019S patients, we obtained fibroblasts from the National Institute of Neurological Disorders (NINDS) repository and from the Telethon Network of Genetics Biobanks (TNGB) for five LRRK2-G2019S and seven additional healthy samples. Patient details including age, sex, MDS-sponsored revision of the Unified Parkinson’s Disease Rating Scale (MDS-UPDRS) score (part III), and Hoehn & Yahr stage are described in Table 1. All patient samples were obtained with appropriate permissions and informed consents in accordance with the ethical guidelines from TUH and from the NINDS repository (USA).

### Fluorescent non-canonical amino acid tagging (FUNCAT)

*L-*azidohomoalanine (AHA) labeling was carried out as previously described^12^. Briefly, skin cells were plated at a density of 50,000 cells per well on round coverslips (1.3 cm diameter). At 48 hours post-plating, cells were washed 1× and incubated with methionine-free Dulbeco’s Modified Essential Medium (DMEM) (Gibco, Thermo Fisher Scientific, Waltham, MA, USA) supplemented with 2 mM Gln 30 min. To label newly synthesized proteins, we incubated cells for 30 min in the methionine-free media containing 1 mM AHA (BCAA005-500, Baseclick, Neuried Germany) (Fig. 1a). Cells were then washed with 1 ml of phosphate-buffered saline (PBS) and fixed by adding 4% paraformaldehyde. Cells were permeablized overnight in *block* (0.25% Triton X100, 0.2% BSA, 5% sucrose, and 10% horse serum in PBS), and washed 5× with 1 mL of 3% BSA in PBS. We carried out cycloaddition of Alexa-488 to AHA-labeled proteins using the Thermo Fisher Scientific kit (catalogue # A10267) according to the manufacturer’s instructions. Nuclei were stained with 1:2000 Hoechst-33342 in PBS. Cells were imaged using a 40× objective and a Leica DMRE microscope with an ORCA C4742-95 CCD camera (Hamamatsu). Images were acquired using identical settings for all samples. Regions of interest (r.o.i.s) of 15 µm^2^ were drawn on cell somas or on an area without cells (background) as indicated in Fig. 1b and intensity values were acquired using ImageJ analysis software. Cell soma r.o.i.s were positioned adjacent to the nucleus which could be detected from Hoechst-33342 DNA stain. Mean intensity values for soma or cell free r.o.i.s from cells with (+AHA, i) or without (-AHA, ii) AHA labeling were acquired as shown (Fig. 1b). Raw mean intensities from background r.o.i.s were subtracted from soma r.o.i.s from + AHA cells. The average soma autofluorescence in -AHA cells was also calculated from multiple cells (3–4 on Fig. 1bii). The average autofluorescence per cell was subtracted from individual soma fluorescence intensities minus background to provide a relative measure of nascent proteins per condition. All analysis was performed by an experimenter that was blinded to the treatment until after the analysis was completed. Statistics were carried out using two-tailed *t*-test and Benjamini Hochberg analysis. The raw data is provided as Supplementary Table 1.

### BONCAT

Patient fibroblasts were plated at a density of 400,000 on 10 cm dishes in 10 ml of Dulbeco’s Modified Essential Medium DMEM supplemented with 10% fetal bovine serum (FBS), 2 mM Gln, penicillin (50 U/ml) streptomycin (50 µg/ml) and incubated at 37 °C in 5% CO_2_ for 6 days (Fig. 2a). Before AHA labeling, medium was replaced with methionine-free RPMI 1640 medium supplemented with 10% dialyzed FBS (A338201, ThermoFisher Scientific; filter-sterilized using a 0.45 µm filter), 2 mM Gln and incubated for 60 min at 37 °C in 5% CO_2_. AHA was added to the medium to a final concentration of 4 mM and cells were incubated for a further 18 h. AHA-labeled cells were collected by scraping, and pelleted at 400 × *g* for 5 min at 4 °C. Cells were lysed with lysis buffer (20 mM N-2-hydroxyethylpiperazine-N′-2-ethanesulfonic acid (HEPES) pH 7.4, 2 mM egtazic acid, 1% sodium dodecyl sulfate (SDS), 50 mM β-glycerophosphate, 1 mM dithiothreitol (DTT), 1 mM Na_3_VO_4_, 1% Triton X-100, 10% Glycerol, 50 mM NaF, 1 mM Benzamidine, 1 µg/ml of Aprotonin, Leupeptin, and Pepstatin 100 µg/ml phenylmethylsulfonyl fluoride (PMSF). DNA was digested with the addition of 250 U/ml Benzonase (#70747, Merck, KGaA, Darmstadt, Germany), and by incubation on ice for 15 min. The resulting lysate was pre-cleared by centrifugation 13,400 × *g* for 5 min at 4 °C, and the supernatant was kept. Protein concentration was determined using a Pierce 660 nm kit with ionic detergent compatibility reagent (Thermo Fisher Scientific Scientific, Inc., Waltham, USA). Supernatants underwent a cyclo-addition reaction to attach AHA-labeled proteins to alkyne agarose using the Click IT cell reaction buffer kit (#C10269, ThermoFisher Scientific). Alkyne agarose (25 μl per sample) was washed with milli-Q and pre-blocked with 2% PVP-40 (Sigma) in *buffer 1* (100 mM Tris pH 8.5, 1% SDS, 250 mM NaCl, 5 mM ethylenediaminetetraacetic acid) and 50 μl of click reaction mix was added to the beads followed by addition of 400 ug of lysate. Samples were incubated for 20 h at room temperature with gentle rotation. Post-click reaction beads were washed with milli-Q and incubated at 70 °C for 15 minutes with 1 ml *buffer 1* supplemented with 1 mM DTT. The beads were washed with *buffer 1* and incubated in dark for 30 min with *buffer 1* containing 2 mM iodoacetamide with gentle rotation.

Following alkylation, the resin was washed with 10 ml of milli-Q followed by 2 × 10 ml washes with *buffer 1* for 30 min each. This was followed by 2 × 10 ml milli-Q washes for 2 min. Resin was then washed with *buffer 2* (100 mM Glycine pH 2.5, 1% SDS). *Buffer 2* was removed by washing 2× with 10 ml of milli-Q for 2 min wash and stored in *buffer 1* until use. SDS was washed away by 1 × 10 ml of 8 M Urea, 1 × 10 ml 20% Isopropanol followed by 1 × 10 ml 20% acetonitrile (ACN) washes. Resin was transferred to a tube in 1 ml of 50 mM Ammonium bicarbonate containing 10% ACN, and then resuspended in 200 µl of the same solution. Digestion was started by adding 0.5 µg of sequencing grade modified trypsin (Promega Corporation, Madison, USA), and proceeded for 16 h at 37 °C with mixing. Digest was harvested, and resin washed with 500 µl milli-Q and combined with digest. The digest was then dried down with a SpeedVac (Thermo Fisher Scientific), and filtered through Whatman Grade 3 filter material and cleaned-up with C18-UltraMicroSpin columns (The Nest Group, Inc., Southborough, USA). The peptides were then dried and dissolved in 0.1% formic acid (FA). Peptide concentration was measured using a Nanodrop nd-1000 (Thermo Fisher Scientific) at 220 nm, and peptide concentrations were normalized before loading for liquid chromatography tandem mass-spectrometry (LC-MS/MS).

### LC-MS/MS for untargeted analysis of AHA labeled samples

A LTQ-Orbitrap XL (Thermo Fisher Scientific) connected to an Eksigent nanoLC plus HPLC system (Eksigent technologies, Dublin, USA) was used. Constant flow of 10 µl/min was maintained for peptide loading onto a pre-column (PepMap 100; Thermo Fisher Scientific). Peptides were then separated on a 10 mm PicoTip™ fused silica emitter 75 µm × 16 cm (New Objective Inc., Woburn, USA), packed in-house with Peprosil C18-AQ resin 3 µm (Dr. Maisch, GmbH, Ammerbuch-Entringen, Germany). LTQ-Orbitrap was set to detect MS spectra at *m/z* 400–2000 with 60,000 resolution at 400 *m/z* in Orbitrap with a mass lock option *m/z* 445.120025. Simultaneously, the top 4 most intense ions in MS/MS were acquired in the LTQ with collision induced dissociation. A dynamic exclusion list restricted to 500 *m/z* values was used for 2 min with a repeat count of 2. Mobile phase A was water/0.1% FA, mobile phase B 100% ACN/0.1% FA. Peptides were separated on linear gradient with mobile phase ramped up to 60 min gradient with 3–35% B was used to elute peptides at constant flow of 300 nl/min.

### Sample preparation for parallel reaction monitoring (PRM) analysis

Fibroblast (passage number 8–10) were cultivated in DMEM as described above and lysed in Laemmli SDS sample buffer (ThermoScientific) Protein concentration was determined using Pierce 660 nm kit with ionic detergent compatibility reagent (Thermo Fisher Scientific), and 12 µg of each sample was loaded on a 12.5% SDS-PAGE gel and run ~1 cm into the resolving gel. Gels were stained with GelCode Blue and samples were excised and cut in to 1 × 1 mm pieces. Gel pieces were destained with 50 mM ammonium bicarbonate in 50% ACN and dried completely in Speedvac. Pieces were rehydrated in 100 mM ammonium bicarbonate containing 10 mM DTT and incubated 1 h at 56 °C. 55 mM iodoacetoamide in equal volume of 100 mM ammonium bicarbonate was added to gel pieces and incubated 1 h at room temperature (RT) in the dark. Following this, excess volume was removed and gel pieces washed with 100 mM ammonium bicarbonate, followed by 100% ACN wash, wash sequence was repeated once. Gels were completely dehydrated with 100% ACN and dried in speedvac. Gel pieces were reswelled in 50 mM ammonium bicarbonate containing trypsin on ice for 30 min (final trypsin:sample ratio 1:50) and incubated overnight at 37 °C. Peptides were extracted 2× with 200 µl 50% ACN containing 0.5% FA, and 1× with 100% ACN. Extracts were pooled and dried completely in the Speedvac and stored at −80 °C until analysis. PRM assay design included peptides that were significantly different in G2019S versus healthy and sporadic versus healthy in the AHA dataset. These were based on a union of significantly altered peptide intensities derived from both raw and relative label-free quantitation (LFQ) intensity values. Synthetic peptides for the chosen targets were ordered from JPT Peptide Technologies (Berlin, Germany) for spectral library generation and scheduling of PRM assay. A spectral library was generated with Mascot (Matrix Science Inc, Boston USA). PRM data was designed and analyzed in Skyline 4.2.0.19072.

### LC-MS/MS – for PRM analysis

was done using a Q-Exactive (Thermo Fisher Scientific) mass spectrometer connected to an Easy-nLC (Thermo Fisher Scientific). Peptides were first loaded on a trapping column and subsequently separated in-line on a 15 cm C18 column (75 μm ID × 15 cm, ReproSil-Pur 5 μm 200 Å C18-AQ, Dr. Maisch HPLC GmbH, Ammerbuch-Entringen, Germany). PRM scans were acquired at 17,500 at 400 *m/z* resolution with a mass lock option *m/z* 445.120025, 1e5 AGC target, 27% collision energy and maximum 50 ms ion accumulation time. 410 ions were monitored in scheduled mode, with each peptide having 3 min analysis window, retention times for the 3 min analysis windows were obtained using synthetic peptides ordered from JPT technologies (Berlin). Mobile phase A was water/0.1% FA and mobile phase B ACN/water (80:20 (v/v)) with 0.1% formic acid in a non-linear gradient. B was first increased from 5 to 21% from 0 to 28 min, then ramped up to 36% from 28 to 50 min an finally to 100% from 50 to 60 min.

### Pre-processing and analysis of AHA LC-MS/MS data

Resulting raw files were searched and quantified with Maxquant 1.6.016 against the UniProt Homo sapiens database (retrieved 12.11.2017) containing isoform information. Carbamidomethylation at cysteine (+57.0215 Da) was set as fixed modification, methionine oxidation (+15.9949 Da) and methionine to AHA substitution (−4.9863 Da) were set as variable modifications for identifications and quantifications. Match between runs and MaxLFQ normalization were turned on. The resulting proteinGroups.txt file was uploaded to Perseus 1.5.8.5, where LFQ intensity values where log2 transformed, and missing values replaced by imputation using Perseus imputation and the normal distribution function. Intensity values were back transformed to original scale, and significantly altered protein intensities from LRRK2-G2019S versus healthy, and sporadic versus healthy were analyzed in R. ROTS (An R package for reproducibility-optimized statistical testing,^65^), and Student’s *t*-test were used with *p* < 0.05 for significant protein group cut-offs. For both comparisons, significant entries were visualized with a heatmap showing sample intensity values, and all entries were visualized with volcano plots showing p-values and Log_2_ fold changes.

### PRM data analysis

Raw files from LC-MS/MS analysis were analyzed in Skyline. In the output, missing values were replaced by 1 and the data was analyzed and visualised using the PhosPiR package in R^20^ with the same cut-offs as described for the AHA data analysis with raw intensity values. Statistical analysis was evaluated using Student’s *t*-test and ROTS^65^. Values with *p* < 0.05 and FDR < 0.05 were counted as significant and included in the list. For both sporadic versus healthy and G2019S versus healthy comparisons, significant results were visualized with a heatmap depicting protein intensities, and all entries were visualized with volcano plots showing p-values and Log_2_ fold changes, using the PhosPir pipeline^20^.

### Western blotting and quantification

Fibroblasts from the TUH sporadic PD cohort and healthy individuals were plated on 10 cm diameter dishes at the same time at a density of 300,000 cells per dish, and grown in DMEM for two days following which they were treated for 16 h with 100 nM MLi-2 or DMSO as indicated. Cells with a passage number of 10-12 were lysed in Laemmli SDS PAGE buffer (ThermoFisher Scientific) and loaded onto a SDS-PAGE gel alongside a normalisation standard which consisted of 5 µg of fibroblast lysate. Proteins were transferred to nitrocellulose membranes using wet transfer as previously described^12^ and incubated at 4 °C overnight with the following antibodies and dilutions; AP2B1 #15690-1-AP (1:4000), YTHDF3 #25537-1-AP (1:3000), ATG9A #67096-1-Ig (1:5000) all from ProteinTech Group Inc. (Rosemont, USA) and EHD1 #MA5-42814 (1:1000), (ThermoFisher Global) and ABHD5 (1:100) (#SC-376931; Santa Cruz Biotechnology, Dallas, USA). Secondary antibodies LiCor IRDye 800Cw goat anti-rabbit and Li-Cor IRDye 680RD donkey anti-mouse (LI-COR Biosciences, Lincoln, USA) were used at a dilution of 1:15,000 in 5% milk in Tris buffered saline (Tris (20 mM), NaCl (150 mM), Tween-20 (0.1% w/v)) and were incubated with the membranes for 1 h followed by washing. Digital images of fluorescence intensities were acquired with the LI-COR Odyssey imaging device.

### Real-time polymerase chain reaction (qPCR) analysis

Relative expression of mRNAs for 23 target genes (listed below) were measured from sporadic and healthy fibroblasts using quantitative real-time PCR (qPCR) as previously described^30^. Primers were designed using PrimerQuest™ Tool from Integrated DNA technologies, and manufactured by Biomers.net (Ulm, Germany). Primers were validated using a QuantStudio™ 12 K Flex Real-Time PCR System from ThermoFisher Scientific. Fibroblast and non-specific template, HEK-293T cDNA were synthesized using a kit (#BIO-65053) from Meridian Bioscience (Finland). For analysis, samples were serially diluted (1.0, 0.5, 0.25, 0.125, 0.0625, 0.03125, and 0.015625) in milli-Q water, in duplicate. The reaction mixture contained 1 µl of diluted cDNA dilution, 0.5 µl of each primer (10 µM) and 5 µl of PowerUp^TM^ SYBR^TM^ Green Master Mix (#A25742; ThermoFisher Scientific). Cycling conditions were initial hold stage of 2 min at 50 °C, initial denaturation for 2 min at 95 °C, followed by 40 cycles consisting of denaturation for 15 s at 95 °C, annealing for 15 s at 60 °C, and 1 minute of extension at 72 °C. The Cq values determination and melt curve analysis was carried out by instrument’s software QuantStudio^TM^ 12 K Flex Software v1.3. Primer efficiencies were determined as qPCR efficiencies calculated from the standard curve slope. Total RNA from patient and healthy fibroblasts was extracted using NucleoSpin RNA Mini kit from Macherey-Nagel (#740955) according to the manufacturer’s protocol. The method included DNase I treatment to degrade contaminating genomic DNA. One microgram of total RNA in a 20 µl reaction was reverse transcribed using SensiFast cDNA synthesis kit from Meridian Bioscience (#BIO-65053). Quantitative real-time PCR was performed on the same instrument with same cycling conditions and software for amplification analysis, as described for primer efficiency determination. The reaction mixture (10 µl) contained 1 µl of 30× diluted cDNA (~1.6 ng), 0.5 µl of each primer (10 µM) and 5 µl of PowerUp™ SYBR™ Green Master Mix (#A25742) (ThermoFisher Scientific). Gene expression levels of 23 target genes relative to GAPDH were measured with accounting for differences in primer efficiencies. The relative gene expression levels were normalized to expression in healthy controls to determine the fold change expression, and compared in a two-tailed Student’s *t*-test.

Primer sequences

**Table:** 

Gene name	Forward primer sequence (5′-3′)	Reverse primer sequence (5′-3′)	Amplicon length (bp)
ATG9A	GATCCACCGGCTTATCAAGTT	CAATACGGAAGGGCAGACATAG	105
EHD1	GATCAGCAGAGGCTATGACTTT	TCCGAGAACTCATCGGAGAT	114
CCDC6	GCAGGAATTTCAGGTCAACAAA	TCCCGTCTCAACTGTTCTAATG	96
SAP18	CGGGTCTTCACCACCAATAA	TTGCATCCATCCAAGTGTAGAT	103
YTHDF3	CTGATGGACAGGCTGGATTT	TGTCAGGTCACCACCAATTT	104
C1orf174	GTTGTGAGTGACTCTCGCTTAG	GGAGCTGCTGTTGGATTCTT	96
ALDH1B1	GTCATGCAGGGTTGGAAACT	CTTGATGAGGGAGGCCAAATAC	111
VASN	GTCTCACCTATCGCAACCTATC	GCATGACACAGACGGAGTAA	126
ABHD5	CTTACTCGCTGAAGTACCCATC	CAAACTGGAATTGGTCTGTCTTG	109
USP7	CCAGGAGAAGGAGTTTGAGAAG	GGCCGAGGATGAGACATATTAC	135
TMEM214	TCTCCCTTTGCCATCACATAC	AAAGTCCAGAAGTGGGAAGAAG	108
JAGN1	TTCCATCGCTCCACTCATTTAT	AACCAAAGAGGAAACGGTAGG	95
GAPDH	TCGGAGTCAACGGATTTGGTC	TGAAGGGGTCATTGATGGCA	96
APOL2	GGATAAGGAGGGCAAGGTAAAG	TATTGCAGGCTCCAGTGTTC	139
CNOT7	TACAGAATGGCACAGGGAATG	CGAGGGATTCAACCAGAGATAAA	131
EPN4	TGCCTAGCAGCAAGTCATC	AGTCAGCAAATCCTCCGAATAA	99
G3BP2	GCAACCTTGAGTGATGGAGTAG	CCTTCAGGAGCCAGAACAAA	104
KHDBS1	CTTGGACCACAAGGGAATACA	CTCTTCCTCCTTGGCTTTGT	105
MGAT1	AGGTCTCAGTCTCACTCTTCT	CCTTCTTCCTTCCTCCTTCTTC	102
NDUFA9	TCACAGATTGTTCCTCCCATTC	TGCATCCGCTCCACTTTATC	117
RPS5	AAGCTCTTTGGGAAGTGGAG	GGCAGGTACTTGGCATACTT	98
RPS15a	CTCACAGGCAGGCTAAACAA	GCGGGATGGAAGCAGATTAT	99

### Functional enrichment analysis

was done using the search tool for the retrieval of interacting genes/proteins (STRING) database^21^. For this, a list of significantly (*p* < 0.05) changing proteins (based on protein data from discovery or PRM analysis as indicated) in LRRK2-G2019S versus healthy and sporadic versus healthy within a given cut-off (described in the legend) was submitted for functional enrichment analysis. The top-ranking enrichments are shown. The strength is defined as the log10(observed/expected) network proteins. FDR represents *p*-values corrected for multiple testing using the Benjamini-Hochberg procedure.

### Reporting summary

Further information on research design is available in the Nature Research Reporting Summary linked to this article.

## Supplementary information

Supplementary information

Supplementary table 1.

Supplementary table 2.

Supplementary table 3.

Supplementary table 4.

Reporting Summary

## Data Availability

The LC-MS/MS data for this study has been uploaded to the Proteomics Identification Database (PRIDE) and can be accessed using the identifier: PXD031144.

## References

[1] Collaborators GPSD (2018). Global, regional, and national burden of Parkinson’s disease, 1990-2016: a systematic analysis for the Global Burden of Disease Study 2016. Lancet Neurol..

[2] Kalia LV, Lang AE (2015). Parkinson’s disease. Lancet.

[3] Berg D (2021). Prodromal Parkinson disease subtypes - key to understanding heterogeneity. Nat. Rev. Neurol..

[4] Titova N, Padmakumar C, Lewis SJG, Chaudhuri KR (2017). Parkinson’s: a syndrome rather than a disease?. J. Neural Transm..

[5] Parnetti L (2019). CSF and blood biomarkers for Parkinson’s disease. Lancet Neurol..

[6] Schilder BM, Navarro E, Raj T (2021). Multi-omic insights into Parkinson’s Disease: from genetic associations to functional mechanisms. Neurobiol. Dis..

[7] Powers ET, Morimoto RI, Dillin A, Kelly JW, Balch WE (2009). Biological and chemical approaches to diseases of proteostasis deficiency. Annu. Rev. Biochem..

[8] Martin I (2014). Ribosomal protein s15 phosphorylation mediates LRRK2 neurodegeneration in Parkinson’s disease. Cell.

[9] Pain S (2019). Regulation of protein synthesis and apoptosis in lymphocytes of Parkinson patients: the effect of dopaminergic treatment. Neurodegener. Dis..

[10] Kim JW (2021). Dysregulated mRNA translation in the G2019S LRRK2 and LRRK2 knock-out mouse brains. eNeuro.

[11] Pallos J, Jeng S, McWeeney S, Martin I (2021). Dopamine neuron-specific LRRK2 G2019S effects on gene expression revealed by translatome profiling. Neurobiol. Dis..

[12] Deshpande P (2020). Protein synthesis is suppressed in sporadic and familial Parkinson’s disease by LRRK2. FASEB J..

[13] Wang Z (2020). Skin α-synuclein aggregation seeding activity as a novel biomarker for Parkinson disease. JAMA Neurol..

[14] Doppler K (2021). Detection of dermal alpha-synuclein deposits as a biomarker for Parkinson’s disease. J. Parkinsons Dis..

[15] Orrù CD (2021). A rapid α-synuclein seed assay of Parkinson’s disease CSF panel shows high diagnostic accuracy. Ann. Clin. Transl. Neurol..

[16] Sinclair E (2021). Validating differential volatilome profiles in Parkinson’s disease. ACS Cent. Sci..

[17] Trapecar M (2021). Human physiomimetic model integrating microphysiological systems of the gut, liver, and brain for studies of neurodegenerative diseases. Sci. Adv..

[18] Dieterich DC, Link AJ, Graumann J, Tirrell DA, Schuman EM (2006). Selective identification of newly synthesized proteins in mammalian cells using bioorthogonal noncanonical amino acid tagging (BONCAT). Proc. Natl Acad. Sci. USA.

[19] tom Dieck S (2015). Direct visualization of newly synthesized target proteins in situ. Nat. Methods.

[20] Hong Y (2022). PhosPiR: an automated phosphoproteomic pipeline in R. Brief. Bioinform..

[21] Szklarczyk D (2021). The STRING database in 2021: customizable protein-protein networks, and functional characterization of user-uploaded gene/measurement sets. Nucleic Acids Res..

[22] Kett LR (2012). LRRK2 Parkinson disease mutations enhance its microtubule association. Hum. Mol. Genet..

[23] Schmidt SH (2019). The dynamic switch mechanism that leads to activation of LRRK2 is embedded in the DFGψ motif in the kinase domain. Proc. Natl Acad. Sci. USA.

[24] Calogero AM, Mazzetti S, Pezzoli G, Cappelletti G (2019). Neuronal microtubules and proteins linked to Parkinson’s disease: a relevant interaction?. Biol. Chem..

[25] Deniston CK (2020). Structure of LRRK2 in Parkinson’s disease and model for microtubule interaction. Nature.

[26] Di Maio R (2018). LRRK2 activation in idiopathic Parkinson’s disease. Sci. Transl. Med..

[27] Melachroinou K (2020). Elevated in vitro kinase activity in peripheral blood mononuclear cells of leucine-rich repeat kinase 2 G2019S carriers: a novel enzyme-linked immunosorbent assay-based method. Mov. Disord..

[28] Puri C, Renna M, Bento CF, Moreau K, Rubinsztein DC (2014). ATG16L1 meets ATG9 in recycling endosomes: additional roles for the plasma membrane and endocytosis in autophagosome biogenesis. Autophagy.

[29] Yen Y-P, Chen J-A (2021). The m6A epitranscriptome on neural development and degeneration. J. Biomed. Sci..

[30] Pfaffl MW (2001). A new mathematical model for relative quantification in real-time RT-PCR. Nucleic Acids Res..

[31] Planken A (2017). Looking beyond the brain to improve the pathogenic understanding of Parkinson’s disease: implications of whole transcriptome profiling of Patients’ skin. BMC Neurol..

[32] Peterson AC, Russell JD, Bailey DJ, Westphall MS, Coon JJ (2012). Parallel reaction monitoring for high resolution and high mass accuracy quantitative, targeted proteomics. Mol. Cell Proteom..

[33] Aebersold R, Burlingame AL, Bradshaw RA (2013). Western blots versus selected reaction monitoring assays: time to turn the tables?. Mol. Cell Proteom..

[34] Madureira M, Connor-Robson N, Wade-Martins R (2020). LRRK2: autophagy and lysosomal activity. Front. Neurosci..

[35] Usmani A, Shavarebi F, Hiniker A (2021). The cell biology of LRRK2 in Parkinson’s disease. Mol. Cell Biol..

[36] Cui Y (2019). Retromer has a selective function in cargo sorting via endosome transport carriers. J. Cell Biol..

[37] Schwartzentruber J (2021). Genome-wide meta-analysis, fine-mapping and integrative prioritization implicate new Alzheimer’s disease risk genes. Nat. Genet..

[38] Xilouri M, Brekk OR, Stefanis L (2016). Autophagy and alpha-synuclein: relevance to Parkinson’s disease and related synucleopathies. Mov. Disord..

[39] Levine B, Kroemer G (2019). Biological functions of autophagy genes: a disease perspective. Cell.

[40] De Tito S, Hervás JH, van Vliet AR, Tooze SA (2020). The golgi as an assembly line to the autophagosome. Trends Biochem. Sci..

[41] Yamano K, Youle RJ (2020). Two different axes CALCOCO2-RB1CC1 and OPTN-ATG9A initiate PRKN-mediated mitophagy. Autophagy.

[42] Saitoh T (2009). Atg9a controls dsDNA-driven dynamic translocation of STING and the innate immune response. Proc. Natl Acad. Sci. USA.

[43] Zavodszky E (2014). Mutation in VPS35 associated with Parkinson’s disease impairs WASH complex association and inhibits autophagy. Nat. Commun..

[44] Bonam SR, Tranchant C, Muller S (2021). Autophagy-lysosomal pathway as potential therapeutic target in Parkinson’s disease. Cells.

[45] Rieske P, Krynska B, Azizi SA (2005). Human fibroblast-derived cell lines have characteristics of embryonic stem cells and cells of neuro-ectodermal origin. Differentiation.

[46] Korecka JA (2019). Mitochondrial clearance and maturation of autophagosomes are compromised in LRRK2 G2019S familial Parkinson’s disease patient fibroblasts. Hum. Mol. Genet..

[47] González-Casacuberta I (2019). Mitochondrial and autophagic alterations in skin fibroblasts from Parkinson disease patients with Parkin mutations. Aging.

[48] Yamaguchi J (2018). Atg9a deficiency causes axon-specific lesions including neuronal circuit dysgenesis. Autophagy.

[49] Kordower JH (2013). Disease duration and the integrity of the nigrostriatal system in Parkinson’s disease. Brain.

[50] Furukawa K (2022). Motor progression and nigrostriatal neurodegeneration in Parkinson disease. Ann. Neurol..

[51] Zou L, Tian Y, Zhang Z (2021). Dysfunction of synaptic vesicle endocytosis in Parkinson’s disease. Front. Integr. Neurosci..

[52] Heaton GR (2020). Sequential screening nominates the Parkinson’s disease associated kinase LRRK2 as a regulator of Clathrin-mediated endocytosis. Neurobiol. Dis..

[53] Wang B (2015). Improved anti-glioblastoma efficacy by IL-13Ralpha2 mediated copolymer nanoparticles loaded with paclitaxel. Sci. Rep..

[54] Fan Y (2018). Interrogating Parkinson’s disease LRRK2 kinase pathway activity by assessing Rab10 phosphorylation in human neutrophils. Biochem. J..

[55] Atashrazm F (2019). LRRK2-mediated Rab10 phosphorylation in immune cells from Parkinson’s disease patients. Mov. Disord..

[56] Fraser KB, Moehle MS, Alcalay RN, West AB, Consortium LC (2016). Urinary LRRK2 phosphorylation predicts parkinsonian phenotypes in G2019S LRRK2 carriers. Neurology.

[57] Wang S (2022). Elevated urinary Rab10 phosphorylation in idiopathic Parkinson disease. Mov. Disord..

[58] Petropoulou-Vathi L (2022). Distinct profiles of LRRK2 activation and Rab GTPase phosphorylation in clinical samples from different PD cohorts. NPJ Parkinsons Dis..

[59] Zeng X (2021). Benefits of iron chelators in the treatment of Parkinson’s disease. Neurochem. Res..

[60] Song LM (2021). Apoferritin improves motor deficits in MPTP-treated mice by regulating brain iron metabolism and ferroptosis. iScience.

[61] Rocha S (2017). Biological implications of differential expression of mitochondrial-shaping proteins in Parkinson’s disease. Antioxidants.

[62] Bender A (2013). TOM40 mediates mitochondrial dysfunction induced by α-synuclein accumulation in Parkinson’s disease. PLoS ONE.

[63] Seo BA (2021). TRIP12 ubiquitination of glucocerebrosidase contributes to neurodegeneration in Parkinson’s disease. Neuron.

[64] Dong MX (2018). Integrated analysis reveals altered lipid and glucose metabolism and identifies NOTCH2 as a biomarker for Parkinson’s disease related depression. Front. Mol. Neurosci..

[65] Elo LL (2009). Optimized detection of differential expression in global profiling experiments: case studies in clinical transcriptomic and quantitative proteomic datasets. Brief. Bioinform.

