# Supplementary material for: Regulators of proteostasis are translationally repressed in fibroblasts from patients with sporadic and LRRK2-G2019S Parkinson’s disease
Source: NPJ Parkinsons Dis. 2023 Feb 6;9:20. doi: 10.1038/s41531-023-00460-w (PMC9902458; doi:10.1038/s41531-023-00460-w)

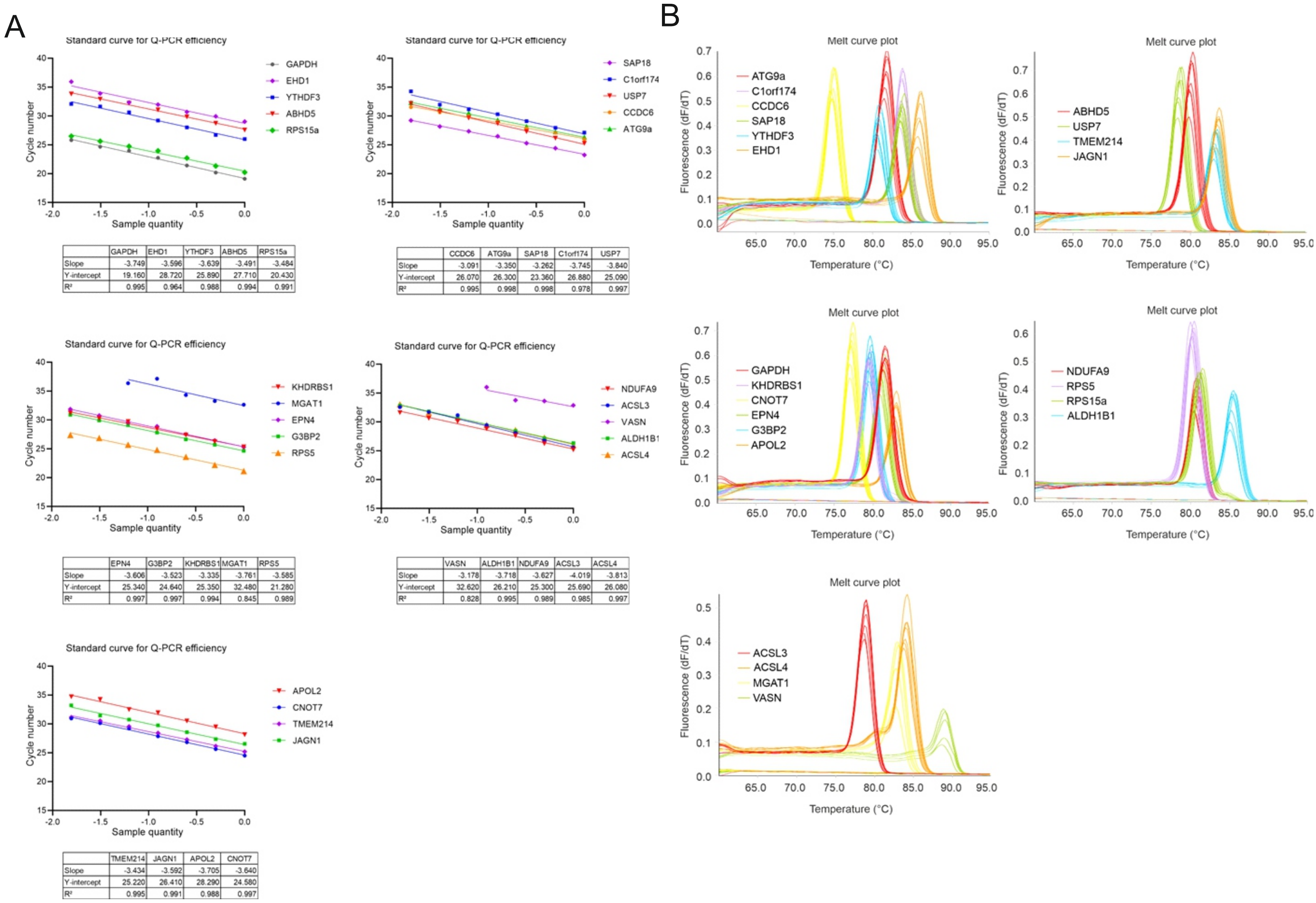

	GAPDH	EHD1	YTHDF3	ABHD5	RPS15a
Slope	-3.749	-3.596	-3.639	-3.491	-3.484
Y-intercept	19.160	28.720	25.890	27.710	20.430
R^2	0.995	0.964	0.988	0.994	0.991

	CCDC6	ATG9a	SAP18	C1orf174	USP7
Slope	-3.091	-3.350	-3.262	-3.745	-3.840
Y-intercept	26.070	26.300	23.360	26.880	25.090
R^2	0.995	0.998	0.998	0.978	0.997

	EPN4	G3BP2	KHDRBS1	MGAT1	RPS5
Slope	-3.606	-3.523	-3.335	-3.761	-3.585
Y-intercept	25.340	24.640	25.350	32.480	21.280
R^2	0.997	0.997	0.994	0.845	0.989

	VASN	ALDH1B1	NDUFA9	ACSL3	ACSL4
Slope	-3.178	-3.718	-3.627	-4.019	-3.813
Y-intercept	32.620	26.210	25.300	25.690	26.080
R^2	0.828	0.995	0.989	0.985	0.997

	TMEM214	JAGN1	APOL2	CNOT7
Slope	-3.434	-3.592	-3.705	-3.640
Y-intercept	25.220	26.410	28.290	24.580
R^2	0.995	0.991	0.988	0.997

Supplementary figure 1. a. Standard curves of quantitative real-time PCR efficiencies for validating primer pairs for 23 genes of interest and GAPDH as a housekeeping gene. Linear fit slope was used for calculating primer efficiencies. Linear fit details, slope value, y-intercept and R2 values, are presented.
b. Melt curve analysis of quantitative real-time PCR for validating primer pairs for 23 genes of interest and GAPDH as a housekeeping gene. Single melt curve peaks confirm amplification of a specific product in HEK-293T cDNA.

Uncropped plots shown on the Figure 7

Target

Actin

ABHD5

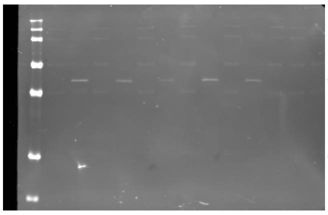

Empty lanes were left between samples for ABHD5 to prevent band migration.

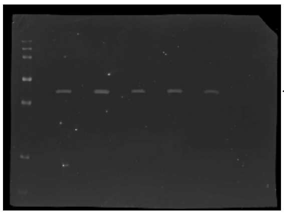

AP2B1

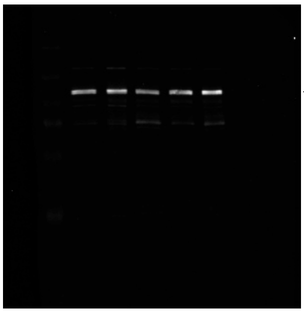

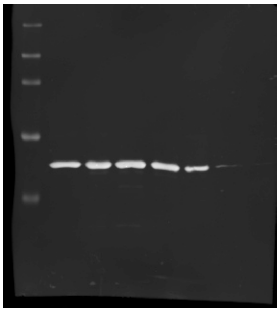

ATG9a

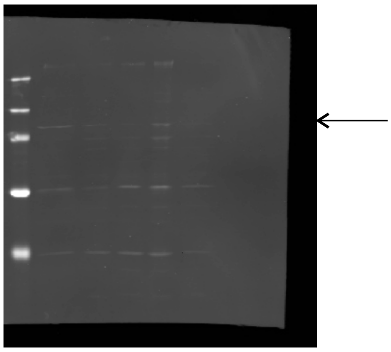

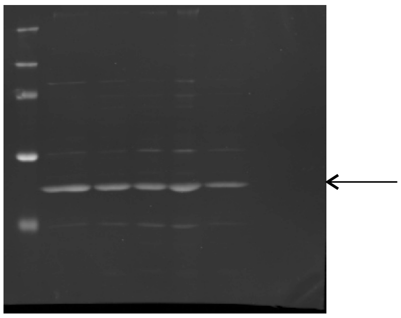

EHD1

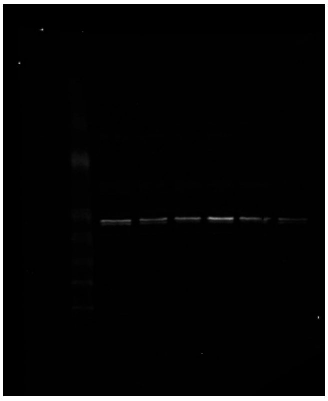

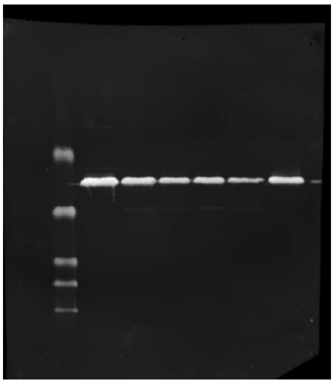

YTHDF3

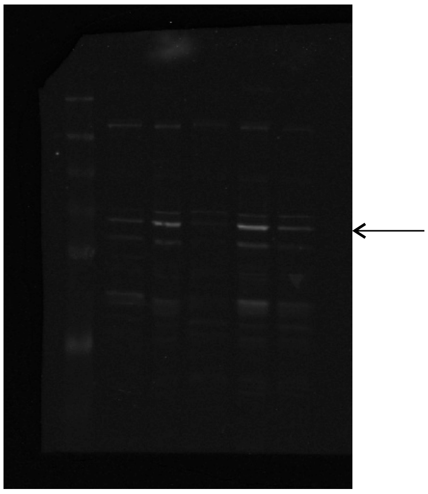

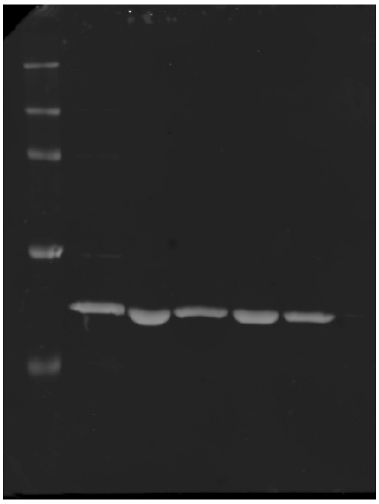

Supplement: Supplementary file 1 — Supplementary information [file 41531_2023_460_MOESM1_ESM.pdf]
