# Supplementary material for: Regulators of proteostasis are translationally repressed in fibroblasts from patients with sporadic and LRRK2-G2019S Parkinson’s disease
Source: NPJ Parkinsons Dis. 2023 Feb 6;9:20. doi: 10.1038/s41531-023-00460-w (PMC9902458; doi:10.1038/s41531-023-00460-w)
Supplement: Supplementary file 6 — Reporting Summary [file 41531_2023_460_MOESM6_ESM.pdf]

nature portfolio

Corresponding author(s): Eleanor Coffey

Last updated by author(s): Jan 13, 2023

Reporting Summary

Nature Portfolio wishes to improve the reproducibility of the work that we publish. This form provides structure for consistency and transparency in reporting. For further information on Nature Portfolio policies, see our Editorial Policies and the Editorial Policy Checklist.

Statistics

For all statistical analyses, confirm that the following items are present in the figure legend, table legend, main text, or Methods section.

n/a	Confirmed	
☐	☒	The exact sample size (*n*) for each experimental group/condition, given as a discrete number and unit of measurement
☒	☐	A statement on whether measurements were taken from distinct samples or whether the same sample was measured repeatedly
☐	☒	The statistical test(s) used AND whether they are one- or two-sided<br>*Only common tests should be described solely by name; describe more complex techniques in the Methods section.*
☐	☒	A description of all covariates tested
☐	☒	A description of any assumptions or corrections, such as tests of normality and adjustment for multiple comparisons
☐	☒	A full description of the statistical parameters including central tendency (e.g. means) or other basic estimates (e.g. regression coefficient) AND variation (e.g. standard deviation) or associated estimates of uncertainty (e.g. confidence intervals)
☐	☒	For null hypothesis testing, the test statistic (e.g. *F*, *t*, *r*) with confidence intervals, effect sizes, degrees of freedom and *P* value noted<br>*Give P values as exact values whenever suitable.*
☒	☐	For Bayesian analysis, information on the choice of priors and Markov chain Monte Carlo settings
☒	☐	For hierarchical and complex designs, identification of the appropriate level for tests and full reporting of outcomes
☒	☐	Estimates of effect sizes (e.g. Cohen's *d*, Pearson's *r*), indicating how they were calculated

Our web collection on statistics for biologists contains articles on many of the points above.

Software and code

Policy information about availability of computer code

Data collection	ThermoFisher Scientific Xcalibur(tm) was used to acquire mass-spectrometry data.
Data analysis	MS data was analysed with MaxQuant 1.6.016 (https://www.maxquant.org/,https://www.nature.com/articles/nbt.1511), Perseus 1.5.8.5(https://www.maxquant.org/, https://www.nature.com/articles/nmeth.3901), Skyline 4.2.0.19072 (https://skyline.ms/project/home/software/Skyline/begin.view, https://academic.oup.com/bioinformatics/article/26/7/966/212410?) andPhosPiR 1.0 (https://github.com/TCB-yehong/PhosPiR, https://academic.oup.com/bib/article/23/1/bbab510/6456296)

For manuscripts utilizing custom algorithms or software that are central to the research but not yet described in published literature, software must be made available to editors and reviewers. We strongly encourage code deposition in a community repository (e.g. GitHub). See the Nature Portfolio guidelines for submitting code & software for further information.

Data

Policy information about availability of data

All manuscripts must include a data availability statement. This statement should provide the following information, where applicable:

- Accession codes, unique identifiers, or web links for publicly available datasets
- A description of any restrictions on data availability
- For clinical datasets or third party data, please ensure that the statement adheres to our policy

The LC-MS/MS data for this study has been uploaded to the Proteomics Identification Database (PRIDE) and can be accessed using the identifier: PXD031144 or with https://www.ebi.ac.uk/pride/archive/projects/PXD031144.

Human research participants

Policy information about studies involving human research participants and Sex and Gender in Research.

Reporting on sex and gender	It was not possible to have balanced gender groups for this study due to sample availability from NINDS and EMB biobanks. Almost all PD samples G2019S and Sporadic were male sex, and healthy individuals were more mixed. However we do not observe any clustering based on the sex, and have indicated the sex on figure heatmaps showing significantly changing proteins.
Population characteristics	Samples are coming from age matched individuals. The samples are coming from three sources, and the most detailed clinical information is coming from the Finnish cohort.
Recruitment	NINDS and EMB cohort samples were provided from the biobanks without detailed information on recruitment. Finnish cohort samples were recruited by professor Valtteri Kaasinen, from patients undergoing diagnosis and treatment at Turku University Hospital.
Ethics oversight	Patient samples were taken with informed consent and the work was approved by Turku University hospital (Permission # T175/2014).

Note that full information on the approval of the study protocol must also be provided in the manuscript.

Field-specific reporting

Please select the one below that is the best fit for your research. If you are not sure, read the appropriate sections before making your selection.

☒ Life sciences ☐ Behavioural & social sciences ☐ Ecological, evolutionary & environmental sciences

For a reference copy of the document with all sections, see nature.com/documents/nr-reporting-summary-flat.pdf

Life sciences study design

All studies must disclose on these points even when the disclosure is negative.

Sample size	Number of samples was determined based on number of publicly available samples and number of individuals willing to participitate in sample collection in Turku, Finland at Turku University Hospital.
Data exclusions	Some MS/MS analyses were excluded based on a low quantitative signal observed after MS/MS analysis. Samples were normalized before loading to mass-spectrometric analysis and it is difficult to pinpoint a cause for these observations.
Replication	Targeted PRM MS/MS results replicated in WB. PRM experiment has unambiguous protein identification opposed to WB with unmeasurable off-target binding to near identical sequences. This is the probable reason why AP2B2 with high sequence identity (83.5%) and near identical size with AP2B1 could have interfered with AP1B1 quantification in WB.
Randomization	Samples were grouped according to diagnosis status: healthy, PD G2019S and sporadic PD. No randomization required or possible due to small number of samples
Blinding	AHA measurement was blinded as the analysis is manual. MS/MS analysis, western blotting and qPCR experiments were not blinded as these are considered unbiased experiments, where all samples are equally treated and it is possible to see from sample output if something is amiss.

Reporting for specific materials, systems and methods

We require information from authors about some types of materials, experimental systems and methods used in many studies. Here, indicate whether each material, system or method listed is relevant to your study. If you are not sure if a list item applies to your research, read the appropriate section before selecting a response.

Materials & experimental systems

n/a	Involved in the study
☐	☒ Antibodies
☐	☒ Eukaryotic cell lines
☒	☐ Palaeontology and archaeology
☒	☐ Animals and other organisms
☒	☐ Clinical data
☒	☐ Dual use research of concern

Methods

n/a	Involved in the study
☒	☐ ChIP-seq
☒	☐ Flow cytometry
☒	☐ MRI-based neuroimaging

Antibodies

Antibodies used

AP2B1 #15690-1-AP (1:4000), YTHDF3 #25537-1-AP (1:3000), ATG9A #67096-1-Ig (1:5000) all from ProteinTech Group Inc. (Rosemont, USA) and EHD1 #MA5-42814 (1:1000), (ThermoFisher Global) and ABHD5 (1:100) (#SC-376931; Santa Cruz Biotechnology, Dallas, USA). Secondary antibodies LiCor IRDye 800Cw goat anti-rabbit and Li-Cor IRDye 680RD donkey anti-mouse (LI-COR Biosciences, Lincoln, USA) were used.

Validation

AP2B1 #15690-1 has been succesfully used in 5 publications for WB with control expriments in the publications. YTHDF3 #25537-1-AP has a control experiments in datasheet and has been used in 34 papers with WB, some of which provide control experiments. ATG9A #67096-1-Ig datasheet shows a clear single band at the correct size for WB. EHD1 #MA5-42814 datasheet shows specificity for human tissues. SC-376931 ABHD5 shows a clear signal at the correct size in the antibody datasheet and has been used for WB in two studies.

Eukaryotic cell lines

Policy information about cell lines and Sex and Gender in Research

Cell line source(s)

THE NINDS HUMAN CELL AND DATA REPOSITORY, "Cell Line and DNA Biobank from Patients Affected by Genetic Diseases," member of the Telethon Network of Genetic Biobanks (project no. GTB12001), funded by Telethon Italy, and Turku University Hospital (TUH). TUH samples work was approved by TUH (Permission # T175/2014).

Authentication

These are primary fibroblasts from patients. No authentitication required.

Mycoplasma contamination

Cells were all tested at Turku Bioscience Centre for mycoplasma and were negative.

Commonly misidentified lines
(See ICLAC register)

This study did not use these.
